# Onboard and External Magnetic Bias Estimation for UAS through CDGNSS/Visual Cooperative Navigation

**DOI:** 10.3390/s21113582

**Published:** 2021-05-21

**Authors:** Federica Vitiello, Flavia Causa, Roberto Opromolla, Giancarmine Fasano

**Affiliations:** Department of Industrial Engineering, University of Naples “Federico II”, P.le Tecchio 80, 80125 Naples, Italy; flavia.causa@unina.it (F.C.); g.fasano@unina.it (G.F.)

**Keywords:** multi-UAV cooperation, calibration, magnetic biases, magnetic declination, Levenberg–Marquardt, error budget analysis, pointing analysis

## Abstract

This paper describes a calibration technique aimed at combined estimation of onboard and external magnetic disturbances for small Unmanned Aerial Systems (UAS). In particular, the objective is to estimate the onboard horizontal bias components and the external magnetic declination, thus improving heading estimation accuracy. This result is important to support flight autonomy, even in environments characterized by significant magnetic disturbances. Moreover, in general, more accurate attitude estimates provide benefits for georeferencing and mapping applications. The approach exploits cooperation with one or more “deputy” UAVs and combines drone-to-drone carrier phase differential GNSS and visual measurements to attain magnetic-independent attitude information. Specifically, visual and GNSS information is acquired at different heading angles, and bias estimation is modelled as a non-linear least squares problem solved by means of the Levenberg–Marquardt method. An analytical error budget is derived to predict the achievable accuracy. The method is then demonstrated in flight using two customized quadrotors. A pointing analysis based on ground and airborne control points demonstrates that the calibrated heading estimate allows obtaining an angular error below 1°, thus resulting in a substantial improvement against the use of either the non-calibrated magnetic heading or the multi-sensor-based solution of the DJI onboard navigation filter, which determine angular errors of the order of several degrees.

## 1. Introduction

The use of Unmanned Aerial Vehicles (UAVs) has become of paramount relevance in the recent years, with an exponential growth of small UAVs. They are currently exploited for many different tasks, including search and rescue [1], load carrying [2], mapping of radiation hotspots [3] and fires [4]. In general, magnetometers play a key role on board small UAVs both as navigation instruments and environment mapping sensors.

### 1.1. Related Work

With regard to navigation, despite some limitations, such as low bandwidth and large measurement noise, magnetometers are typically used in outdoor flight operations to bound the error in heading estimation, unless tactical-grade gyros (accurate enough to sense Earth rotation rate) are exploited. Magnetic measurements enable relatively coarse heading estimates, which depend on intrinsic sensors limitations, but above all on disturbances from onboard (e.g., electric rotors and electronic systems) and external sources (e.g., large metal infrastructures). Indeed, such disturbances may significantly alter the direction of the magnetic field and lead to highly inaccurate heading estimates, which may even compromise flight safety if position control is implemented with GNSS (Global Navigation Satellite Systems) information used in feedback. In these scenarios, one possible solution for magnetic-independent heading estimation is given by dual GNSS antenna architectures [5], though the dependence of heading accuracy on antenna baseline, and the additional weight and complexity, may pose challenges for small flight platforms. If magnetic information needs to be exploited, accurate calibration and estimation of onboard and external biases is the key to enable effective compensation of disturbances. State-of-the-art magnetic calibration methods model the intrinsic error sources with an ellipsoid error model whose parameters are estimated by applying a non-linear optimization process [6,7]. Such a procedure, which requires in input a set of measurements collected in different pointing conditions of the sensor’s axes to properly sample the ellipsoid surface, may encounter challenges when applied during UAV flight operations. Recent references addressing magnetometer calibration, but dealing only with estimation of onboard disturbances, can be found in [8,9]. Other recent approaches also focus on the idea to exploit the variations of the external magnetic field also as a source of positioning information: in [10], a magnetic-based simultaneous location and mapping (SLAM) approach has been studied as an alternative to the usual GNSS-based navigation. Such work is mainly focused on the analysis of a magnetic-based navigation concept which does not rely on prior Earth magnetic anomaly field maps.

With regard to the possibility to use magnetometers for environmental mapping purposes, UAV-based magnetometry is gaining increasing popularity. For instance, the feasibility and effectiveness of using a small fixed-wing UAV for aeromagnetic missions in remote areas, such as the Bransfield Basin in Antarctica, was shown in [11]. Specifically, a three-axis fluxgate magnetometer allowed generating a magnetic anomaly map. A similar idea is shown in [12], where an eight-rotors copter (DJI S1000+) with a cesium-vapor magnetometer attached with ropes at a three-meters distance below the UAV (to minimize the effects of onboard disturbances) has been used for archaeological purposes or in [13], where both rotorcraft and fixed-wing UAVs have been used to map the magnetic anomaly of outcrops in a mining district in Finland, showing results which finely compete with those achievable using ground-based sensors, but within a shorter time interval and with a smaller cost. Recent works propose more compact systems with magnetometers installed in closer proximity with respect to the drone frame [14]. In these cases, the need for accurate magnetic calibration also arises as a prerequisite for effective measurement processing.

In this framework, the possibility to exploit cooperation between multiple UAVs can be a powerful tool. In previous works [15,16] the authors proposed a cooperative approach that combines differential GNSS and vision-based tracking between a “chief” and one or more “deputy” drones, to provide accurate magnetic- and inertial-independent attitude information for the chief UAV. The possibility to attain magnetic-independent and drift-free heading information paves the way for the exploitation of the approach for magnetic calibration. The authors proposed in [17,18] a cooperative technique for magnetic calibration in operating flight conditions that exploits differential GNSS and vision data gathered by means of a single deputy UAV. The key idea was to exploit cooperative navigation measurements collected at different heading angles for the chief. The approach was aimed at estimating onboard biases and the problem was formulated as a non-linear system of equations, which was solved by using the Levenberg–Marquardt (LM) iterative method [19].

### 1.2. Paper Contribution

Following the line of research in [13,14,15,16], this paper aims at further improving accuracy in the magnetic heading estimation process by tackling both internal and external magnetic disturbances. Such a result is relevant both to enable reliable autonomous flight in scenarios characterized by significant magnetic disturbances and to ensure more accurate attitude estimation, which provide benefits for georeferencing and mapping applications. The main innovative points can be listed as follows:

- The problem is reformulated to simultaneously compute both the onboard magnetic biases and the external magnetic declination.
- A theoretical error budget is derived that allows predicting the calibration uncertainty as a function of chief–deputy geometries, differential positioning and visual tracking accuracy, and magnetometers sensitivity.
- The approach is tested in flight exploiting accurate carrier phase differential GNSS (CDGNSS) measurements.
- Improvement of heading accuracy is demonstrated by a pointing analysis based on ad hoc Ground and Airborne Control Points.

The paper is structured as follows. The theoretical framework and the mathematical procedure used for magnetic bias estimation is reported in Section 2, whereas the derivation of the associated error budget and its theoretical performance are presented in Section 3. Section 4 contains the experimental setup used for data collection. Experimental results and the performance of the proposed methodology are reported in Section 5. Finally, conclusions and possible following studies are drawn in Section 6.

## 2. Cooperative Magnetic Calibration Method

### 2.1. Nomenclature

Before detailing the adopted methodology, this chapter defines some conventions and symbols that will be used hereafter in the manuscript. Matrices, vectors, and scalars are defined with capital (*A*), bold (**a**), and italic (*a*) font, respectively. The quantity $a^{i}$ indicates a vector in the frame *i*. Camera (CRF), Body (BRF), Body Stabilized (BSRF) and locally levelled North East and Down (NED) reference frames are accounted for in this manuscript. Letters *c*, *b*, *s*, and *n* are used as superscript symbols to indicate CRF, BRF, BSRF and NED frames, respectively. The *k*-th component of the $a^{i}$ vector is indicated as $a_{h}^{i}$, with *h* = *x*, *y*, *z*. For the sake of clarity, *N*, *D* and *E* subscripts are used when the NED frame is accounted for. A vector $a^{i}$ measured by an instrument *m* is indicated by ${\left.a_{}^{i}\right|}_{m}$.

The rotation matrix $M_{i}^{l}$ defines the transformation from frame *i* to frame *l*, such as $a^{l}=M_{i}^{l}a^{i}$. Attitude angles (heading *ψ*, pitch *θ* and roll *φ*), are defined as the 321-sequence associated to the rotation matrix from NED to BRF, i.e., $M_{n}^{b}$. Indicating with *M*_α_ the elementary rotation of angle α, the NED to BRF rotation matrix can be written as:

(1) $$M_{n}^{b}=M_{\varphi }M_{\theta }M_{\psi }\;M_{\varphi }=[\begin{array}{ccc} 1 & 0 & 0\\ 0 & \cos\left(\varphi \right) & \sin\left(\varphi \right)\\ 0 & -\sin\left(\varphi \right) & \cos\left(\varphi \right) \end{array}];M_{\theta }=[\begin{array}{ccc} \cos\left(\theta \right) & 0 & -\sin\left(\theta \right)\\ 0 & 1 & 0\\ \sin\left(\theta \right) & 0 & \cos\left(\theta \right) \end{array}];M_{{}_{\psi }}=[\begin{array}{ccc} \cos\left(\psi \right) & \sin\left(\psi \right) & 0\\ -\sin\left(\psi \right) & \cos\left(\psi \right) & 0\\ 0 & 0 & 1 \end{array}]$$

*I_a_* and 0*_a_* indicate an identity and a zeros matrix of size *a* × *a,* respectively. The error on a quantity **a** is indicated as $\delta a=\hat{a}-a$, which is the difference between the predicted, indicated with a hat (ˆ), and true quantity. The attitude error $\rho ={\left[\begin{array}{ccc} \rho_{N} & \rho_{E} & \rho_{D} \end{array}\right]}^{T}$ expressed in NED frame is composed of horizontal, i.e., *ρ_E_* and *ρ_N_*, and vertical, i.e., (*ρ*_D_), components. It connects the predicted and true NED to BRF rotation matrix with $M_{n}^{b}={\hat{M}}_{n}^{b}(I-[\rho \times ])$, where [×] is the operator yielding the skew symmetric matrix of the vector in the brackets. The Standard Deviation (STD) of scalar and vectorial quantities associated to the measurement instrument *m* are reported as the scalar *σ_m_* and the vector **Σ***_m_*, respectively. The derivative of a *a* × 1 vector **a** with respect to a *b* × 1 vector **b** is a *a* × *b* matrix indicated with ∂a/∂b. The operator that extracts the diagonal from the matrix *A* is indicated as *Diag* (*A*). It returns a vector **v**, while *diag* (**v**) is the operator returning a diagonal matrix, whose elements are the components of the vector **v**.

### 2.2. Methodology

The Earth’s magnetic field (**H**) components along the BRF directions can be measured by a three-axes magnetometer. From these measurements, the magnetic heading angle can be easily estimated by exploiting the sensed horizontal components of the Earth’s magnetic field as referred to the BSRF, which is obtained as a projection of BRF in the local horizontal north–east plane through a combined pitch (ϑ) and roll (φ) rotation as shown in Equation (2).

(2) $$H^{s}={\left[M_{\varphi }M_{\theta }\right]}^{-1}H^{b}$$

The resulting magnetic heading can be therefore expressed as in Equation (3), where *d_m_* is the local magnetic declination, i.e., the angle between the local magnetic (*N_m_*) and geographical (*N*) north directions.

(3) $$\psi_{m}=tg^{-1}(-\frac{H_{y}^{s}}{H_{x}^{s}})+d_{m}$$

However, onboard electric devices, such as the electric rotors, introduce a disturbance on the quantity sensed by the magnetometers. This disturbance can be modelled as a bias, Δ**H**, constant in BRF, which induces an error in the estimation of the Earth’s magnetic field direction. Under the assumption of small roll and pitch rotations, Δ**H** can be assumed to be constant in BSRF, and the effect of the vertical component of Δ**H** in BRF ($\Delta H_{z}^{b}$) on heading estimation can be considered negligible. This assumption is considered in this work since the main interest lies in improving heading and magnetic declination accuracy, without aiming at full 3d calibration.

**Figure 1:**
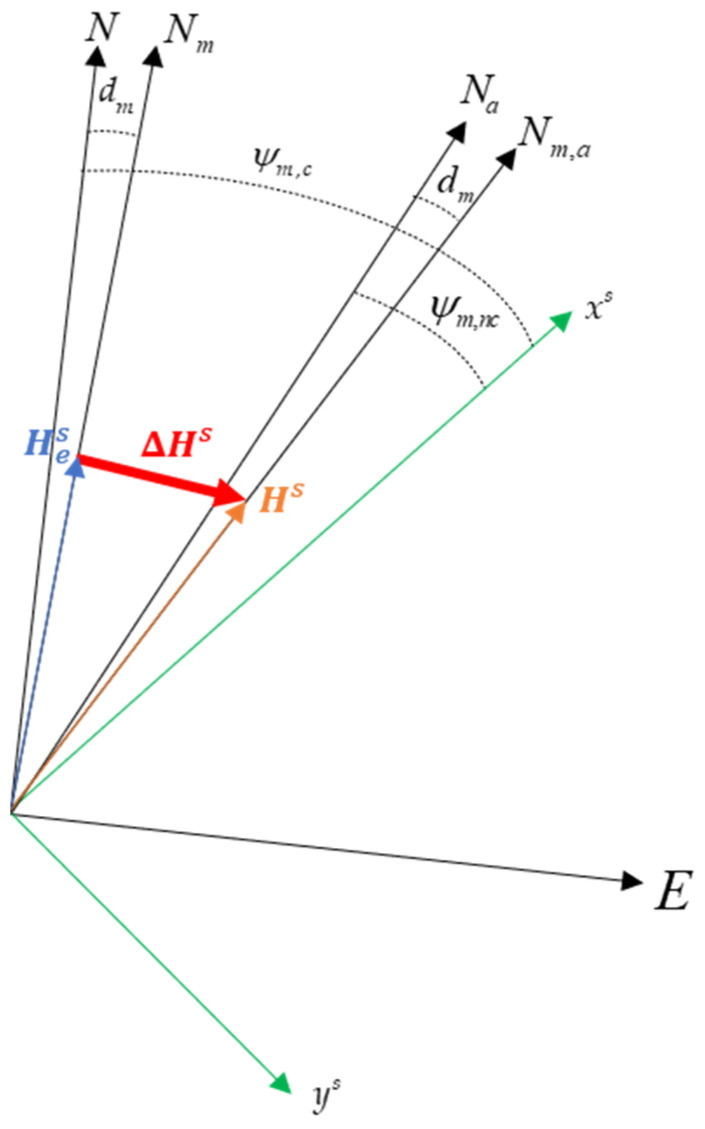
Graphic illustration of the internal magnetic bias effect on the magnetic heading angle estimation.

A graphic illustration of the studied problem can be found in Figure 1, which clearly shows how the magnetic field vector estimated by magnetometers and projected in BSRF, i.e., **H***^s^*, can be expressed as the sum of the true Earth magnetic field vector in BSRF ($H_{e}^{s}$), which is aligned with the N_*m*_ direction, and the internal magnetic biases vector in the same reference frame (Δ**H***^s^*). The effect of such biases can be equivalently seen as an apparent shift of the magnetic and geographic north directions, respectively, to *N_m,a_* and *N_a_*, as shown in Figure 1, thus resulting in the heading angle expressed in Equation (3), which will be referred to as “non-calibrated” ($\psi_{m,nc}$), to be ill-defined. A more accurate angle, referred to as “calibrated” ($\psi_{m,c}$), can then be computed if the in-plane components of Δ**H***^s^* are estimated and removed from its formulation, as expressed in Equation (4).

(4) $$\psi_{m,C}=tg^{-1}(\frac{-H_{y}^{s}+\Delta H_{y}^{s}}{H_{x}^{s}-\Delta H_{x}^{s}})+d_{m}$$

The calibration strategy described in this paper takes its roots from the previous work illustrated in [18]. As a matter of fact, the bias components $\Delta H_{x}^{s}$ and $\Delta H_{y}^{s}$ are computed by using the same iterative least-squares minimization (LM) procedure. However, the magnetic declination at the flight location is added to the unknowns of the problem. Hence, the proposed approach can be used to correct both “onboard” bias and external disturbances by means of multi-UAV cooperation.

The iterative procedure is based on the minimization of the residual obtained as the difference between the unit vector representing the chief–deputy Line Of Sight (LOS) in NED as reported by CDGNSS data and projected in CRF (${\left.u_{}^{c}\right|}_{cdgnss}$), and the same quantity as estimated by visual-based techniques (${\left.u_{}^{c}\right|}_{vision}$). This can be mathematically put as in Equation (5).

(5) $$r={u^{c}|}_{vision}-{u^{c}|}_{cdgnss}=0$$

Visual-based detection and tracking techniques [20] can be used to determine the deputy position in the images collected by the camera on board the chief. Hence, the LOS in CRF is obtained from the pixel coordinates thanks to the intrinsic camera parameters estimated by offline camera calibration. On the other hand, the CDGNSS-based estimate of the LOS in CRF can be obtained by rotating the unit vector corresponding to the CDGNSS relative position vector (${\left.u_{}^{n}\right|}_{cdgnss}$) from NED to BRF, and then from BRF to CRF.

These rotations are achieved by multiplying the vector with the matrices $M_{n}^{b}$ and $M_{b}^{c}$. The misalignment angles between BRF and CRF (determining $M_{b}^{c}$), also referred to as extrinsic camera-IMU rotational parameters, can be computed by the camera offline calibration procedure [21]. More in general, the lever arm of the GNSS antenna with respect to the camera installation on the chief could be considered. However, if the offset is small, the resulting effect is negligible. The final cost vectorial function ***r*** can now be expressed in terms of the unknowns as written in Equation (6).

(6) $$r=f(\Delta H_{x}^{s},\Delta H_{y}^{s},d_{m})={u^{c}|}_{vision}-M_{b}^{c}M_{n}^{b}{u^{n}|}_{cdgnss}$$

This calibration technique fully relies on the availability of GNSS data for both platforms and on the deputy visibility with respect to the chief, thus implying that the two vehicles must fly under nominal GNSS coverage conditions and that the deputy must fall in the Field of View (FOV) of the chief camera. For this reason, as a general condition, chief and deputy should be kept facing each other during the flight, if equipped with strapdown frontal-looking cameras. Moreover, the accuracy of the calibration technique is affected by the inter-UAV distance as discussed in detail in Section 3.1. As a rule of thumb, accurate calibration requires a minimum distance of a few tens of meters.

**Figure 2:**
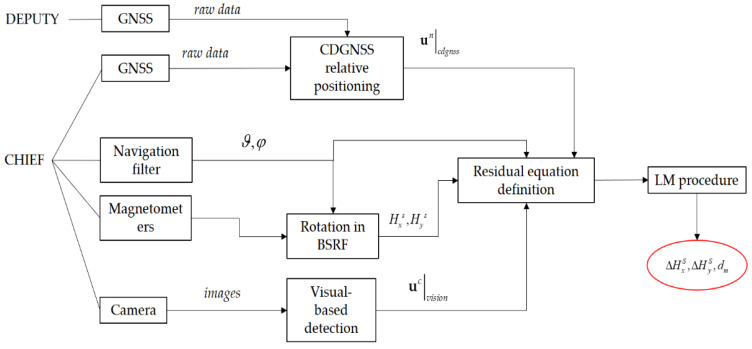
Scheme describing the working principle of the proposed magnetic calibration strategy.

An additional condition for the proposed calibration procedure, is the need to ensure synchronization of GNSS data (from both the chief and deputy) and camera images, thus building a correspondence between the two vectors used in Equation (6). In this respect, accurate synchronization is performed by time-tagging both chief and deputy data with GNSS time. Clearly, to ensure observability of the problem’s unknowns, Equation (6) must be written considering a set of k camera frames. This results in a 3*k* × 1 residual vector ***r*** which can be minimized by applying the LM algorithm. The proposed approach is summarized by the scheme in Figure 2.

Rather than computing the vectorial cost function as Equation (6) states, an equivalent scalar chi-squared function (χ^2^) is computed, and its minimization is carried-out. This function is expressed in Equation (7), where the *W* matrix is the 3*k* × 3*k* block diagonal weight matrix associated to each residual. Such a matrix is shown in Equation (8), where *W*_1_, …, *W_k_* are the 3 × 3 weight diagonal matrices associated to each of the *k* frames and defined, for the *i*-th frame, as $W_{i}=diag(\frac{1}{Diag(R_{cdgnss}^{c}+R_{vision})})$. Here, $R_{cdgnss}^{c}$ and $R_{vision}$ are the covariance matrices associated to CDGNSS and visual measurements expressed in the same reference frame of the residual, i.e., CRF. Detailed derivation of these quantities is reported in Section 3.

(7) $$\chi^{2}(\Delta H_{x}^{s},\Delta H_{y}^{s},d_{m})\;=r^{T}Wr$$

(8) $$W\;=\;\left[\begin{array}{ccccc} W_{1} & 0 & & \cdots & 0\\ 0 & W_{2} & 0 & \cdots & 0\\ \vdots & \ddots & \ddots & \ddots & \vdots \\ 0 & & \cdots & 0 & W_{k} \end{array}\right]$$

At each new iteration, a correction of the unknown quantities, i.e., $h_{LM}$, is added to their value as computed at the previous step. This quantity is expressed in Equation (9), where *J* is the Jacobian matrix representing the derivatives of the cost function **r** with respect to the three unknowns identified by the vector $x={\left[\begin{array}{ccc} \Delta H_{x}^{s} & \Delta H_{y}^{s} & d_{m} \end{array}\right]}^{T}$, *λ* is the LM damping parameter representing how close the procedure is getting to the gradient descent or the Gauss–Newton methods, and $diag(Diag(J^{T}WJ))$ is the matrix that only contains the diagonal of the $J^{T}WJ$ matrix.

(9) $$h_{LM}={\left(J^{T}WJ+\lambda \cdot diag(Diag(J^{T}WJ))\right)}^{-1}J^{T}Wr$$

The iterative procedure is either stopped when one of the convergence criteria for the LM procedure, as listed in Equation (10), is met or when the maximum number of iterations is reached.

(10) $$\begin{array}{l} \max(\left|J^{T}Wr\right|)<\varepsilon_{1}\\ \max(\left|\frac{h_{LM}}{x}\right|)<\varepsilon_{2}\\ \frac{\chi^{2}}{k-1}<\varepsilon_{3} \end{array}$$

## 3. Error Budget

Quantification of uncertainty in estimating magnetometer biases and magnetic declination plays a fundamental role in understanding the lower error bounds for the calibration technique, and it can be useful both for system design and flight planning. This section aims at explicitly deriving magnetometer biases and external declination STD, directly from equations reported in Section 2.

Using the error definition given in Section 2.1, the error form of Equation (5) can be expressed as:

(11) $$r={-\delta u^{c}|}_{vision}+M_{b}^{c}{\hat{M}}_{\varphi }{\hat{M}}_{\theta }{\hat{M}}_{\psi_{m,C}}{\delta u^{n}|}_{cdgnss}+M_{b}^{c}{\hat{M}}_{\varphi }{\hat{M}}_{\theta }{\hat{M}}_{\psi_{m,C}}[{{\hat{u}}^{n}|}_{cdgnss}\times ]\rho$$

Decomposing **ρ** in horizontal and vertical error and grouping ${\hat{M}}_{n}^{b}={\hat{M}}_{\varphi }{\hat{M}}_{\theta }{\hat{M}}_{\psi_{m,C}}$, Equation (11) becomes:

(12) $$r=-{\delta u^{c}|}_{vision}+M_{b}^{c}{\hat{M}}_{n}^{b}{\delta u^{n}|}_{cdgnss}+M_{b}^{c}{\hat{M}}_{n}^{b}[{{\hat{u}}^{n}|}_{cdgnss}\times ][\begin{array}{c} \rho_{N}\\ \rho_{E}\\ 0 \end{array}]+M_{b}^{c}{\hat{M}}_{n}^{b}[\begin{array}{c} {{\hat{u}}_{E}^{n}|}_{cdgnss}\rho_{D}\\ -{{\hat{u}}_{N}^{n}|}_{cdgnss}\rho_{D}\\ 0 \end{array}]$$

*ρ_D_* is the rotation error along the down axis which corresponds to the heading error, i.e., *δψ_m,c_*. The latter can be derived from Equation (4), observing that $\psi_{m,C}=f(H_{x}^{s},H_{y}^{s},\Delta H_{x}^{s},\Delta H_{y}^{s},d_{m})$. Hence, expanding at first order:

(13) $$\begin{array}{c} \rho_{D}=\delta \psi_{m,C}=\frac{\partial \psi_{m,C}}{\partial H_{x}^{s}}\delta H_{x}^{s}+\frac{\partial \psi_{m,C}}{\partial H_{y}^{s}}\delta H_{y}^{s}+\frac{\partial \psi_{m,C}}{\partial \Delta H_{x}^{s}}\delta \Delta H_{x}^{s}+\frac{\partial \psi_{m,C}}{\partial \Delta H_{y}^{s}}\delta \Delta H_{y}^{s}+\delta d_{m}\\ \frac{\partial \psi_{m,C}}{\partial H_{x}^{s}}=-\frac{1}{1+{\left(\frac{-H_{y}^{s}+\Delta H_{y}^{s}}{H_{x}^{s}-\Delta H_{x}^{s}}\right)}^{2}}\frac{-H_{y}^{s}+\Delta H_{y}^{s}}{{\left(H_{x}^{s}-\Delta H_{x}^{s}\right)}^{2}}\\ \frac{\partial \psi_{m,C}}{\partial H_{y}^{s}}=-\frac{1}{1+{\left(\frac{-H_{y}^{s}+\Delta H_{y}^{s}}{H_{x}^{s}-\Delta H_{x}^{s}}\right)}^{2}}\frac{1}{H_{x}^{s}-\Delta H_{x}^{s}}\\ \frac{\partial \psi_{m,C}}{\partial \Delta H_{x}^{s}}=\frac{1}{1+{\left(\frac{-H_{y}^{s}+\Delta H_{y}^{s}}{H_{x}^{s}-\Delta H_{x}^{s}}\right)}^{2}}\frac{-H_{y}^{s}+\Delta H_{y}^{s}}{{\left(H_{x}^{s}-\Delta H_{x}^{s}\right)}^{2}}\\ \frac{\partial \psi_{m,C}}{\partial \Delta H_{y}^{s}}=\frac{1}{1+{\left(\frac{-H_{y}^{s}+\Delta H_{y}^{s}}{H_{x}^{s}-\Delta H_{x}^{s}}\right)}^{2}}\frac{1}{H_{x}^{s}-\Delta H_{x}^{s}} \end{array}$$

Substituting Equation (13) in Equation (12), the residual can be expressed as a function of the magnetometer biases as:

(14) $$r=-{\delta u^{c}|}_{vision}+M_{b}^{c}{\hat{M}}_{n}^{b}{\delta u^{n}|}_{cdgnss}+M_{b}^{c}{\hat{M}}_{n}^{b}[{\delta {\hat{u}}^{n}|}_{cdgnss}\times ][\begin{array}{c} \rho_{N}\\ \rho_{E}\\ 0 \end{array}]+M_{b}^{c}{\hat{M}}_{n}^{b}[\begin{array}{c} {{\hat{u}}_{E}^{n}|}_{cdgnss}\\ -{{\hat{u}}_{N}^{n}|}_{cdgnss}\\ 0 \end{array}](\frac{\partial \psi_{m,C}}{\partial \Delta H_{x}^{s}}\delta \Delta H_{x}^{s}+\frac{\partial \psi_{m,C}}{\partial \Delta H_{y}^{s}}\delta \Delta H_{y}^{s}+\delta d_{m})+\;+M_{b}^{c}{\hat{M}}_{n}^{b}[\begin{array}{c} {{\hat{u}}_{E}^{n}|}_{cdgnss}\\ -{{\hat{u}}_{N}^{n}|}_{cdgnss}\\ 0 \end{array}](\frac{\partial \psi_{m,C}}{\partial H_{x}^{s}}\delta H_{x}^{s}+\frac{\partial \psi_{m,C}}{\partial H_{y}^{s}}\delta H_{y}^{s})$$

From Equation (14), one can derive an expression for the residual **r**, such as

(15) r=Jδx+w

where *δ***x** is the vector including the error on the unknown variables, $\delta x={\left[\begin{array}{ccc} \delta \Delta H_{x}^{s} & \delta \Delta H_{y}^{s} & \delta d_{m} \end{array}\right]}^{T}$, *J* is the measurement matrix and **w** is the residual error, which is assumed to be a Gaussian zero-mean with covariance *R*. Therefore, Equation (14) is rewritten in the form of Equation (15) as:

(16) $$\begin{array}{c} r=\underset{J}{\underbrace{M_{b}^{c}{\hat{M}}_{n}^{b}\left[\begin{array}{ccc} {\left.{\hat{u}}_{E}^{n}\right|}_{cdgnss}\frac{\partial \psi_{m,C}}{\partial \Delta H_{x}^{s}} & {\left.{\hat{u}}_{E}^{n}\right|}_{cdgnss}\frac{\partial \psi_{m,C}}{\partial \Delta H_{y}^{s}} & {\left.{\hat{u}}_{E}^{n}\right|}_{cdgnss}\\ -{\left.{\hat{u}}_{N}^{n}\right|}_{cdgnss}\frac{\partial \psi_{m,C}}{\partial \Delta H_{x}^{s}} & -{\left.{\hat{u}}_{N}^{n}\right|}_{cdgnss}\frac{\partial \psi_{m,C}}{\partial \Delta H_{y}^{s}} & -{\left.{\hat{u}}_{N}^{n}\right|}_{cdgnss}\\ 0 & 0 & 0 \end{array}\right]}}\delta x\\ w=-\delta {\left.u_{}^{c}\right|}_{vision}+\underset{\frac{\partial r}{\partial u^{n}}}{\underbrace{M_{b}^{c}{\hat{M}}_{n}^{b}}}\delta {\left.u_{}^{n}\right|}_{cdgnss}+\underset{\frac{\partial r}{\partial \varepsilon }}{\underbrace{M_{b}^{c}{\hat{M}}_{n}^{b}\left[{\left.{\hat{u}}_{}^{n}\right|}_{cdgnss}\times \right]}}\left[\begin{array}{c} \rho_{N}\\ \rho_{E}\\ 0 \end{array}\right]+\underset{\frac{\partial r}{\partial H_{h}}}{\underbrace{M_{b}^{c}{\hat{M}}_{n}^{b}\left[\begin{array}{cc} {\hat{u}}_{y}^{n}\frac{\partial \psi_{m,C}}{\partial H_{x}^{s}} & {\hat{u}}_{y}^{n}\frac{\partial \psi_{m,C}}{\partial H_{y}^{s}}\\ -{\hat{u}}_{x}^{n}\frac{\partial \psi_{m,C}}{\partial H_{x}^{s}} & -{\hat{u}}_{x}^{n}\frac{\partial \psi_{m,C}}{\partial H_{y}^{s}}\\ 0 & 0 \end{array}\right]}}\left[\begin{array}{c} \delta H_{x}^{s}\\ \delta H_{y}^{s} \end{array}\right]\\ R=\;R_{vision}+\underset{R_{cdgnss}^{c}}{\underbrace{\frac{\partial r}{\partial u^{n}}R_{cdgnss}{\frac{\partial r}{\partial u^{n}}}^{T}}}+\frac{\partial r}{\partial \varepsilon }\left[\begin{array}{ccc} \rho_{N}{}^{2} & 0 & 0\\ 0 & \rho_{E}{}^{2} & 0\\ 0 & 0 & 0 \end{array}\right]{\frac{\partial r}{\partial \varepsilon }}^{T}+\frac{\partial r}{\partial H_{h}}\left[\begin{array}{cc} {\left(\delta H_{x}^{s}\right)}^{2} & 0\\ 0 & {\left(\delta H_{y}^{s}\right)}^{2} \end{array}\right]{\frac{\partial r}{\partial H_{h}}}^{T} \end{array}$$

The covariance on CDGNSS unit vector (*R_cdgnss_*) is obtained by transforming the estimated CDGNSS baseline’s STD vector ($\Sigma_{cdgnss}$) in unit vector covariance, as

(17) $$R_{cdgnss}=\frac{\partial u^{n}}{\partial v^{n}}diag\left(\Sigma_{cdgnss}\right){\frac{\partial u^{n}}{\partial v^{n}}}^{T}$$

where $\partial u^{n}/\partial v^{n}$ is the derivative of a unit vector **u** with respect to its associated vector **v**. The norm of this matrix reduces by increasing the norm of **v**, making *R_cdgnss_* smaller. The covariance on CDGNSS measurements can be expressed in CRF by using:

(18) $$R_{cdgnss}^{c}=\frac{\partial r}{\partial u^{n}}R_{cdgnss}{\frac{\partial r}{\partial u^{n}}}^{T}$$

*R_vision_*, i.e., the covariance on visual measurements, can be derived by converting the error on azimuth and elevation angles estimated by the camera in unit vector error. The camera angular STD, i.e., σ*_vision_* is at best equal to the IFOV (unless sub-pixel performance is achieved by the visual tracking system) and is assumed to be the same both horizontally and vertically. Naming $\partial u^{c}/\partial Az$ and $\partial u^{c}/\partial El$ the derivative of the camera unit vector with respect to azimuth and elevation, respectively, *R_vision_* is:

(19) $$R_{vision}=\left[\begin{array}{cc} \frac{\partial u^{c}}{\partial Az} & \frac{\partial u^{c}}{\partial El} \end{array}\right]\left[\begin{array}{cc} \sigma_{vision}^{2} & 0\\ 0 & \sigma_{vision}^{2} \end{array}\right]{\left[\begin{array}{cc} \frac{\partial u^{c}}{\partial Az} & \frac{\partial u^{c}}{\partial El} \end{array}\right]}^{T}$$

Estimating the covariance on *δ***x**, $\mathrm{cov}(\delta x)={\left(J^{T}R^{-1}J\right)}^{-1}$, requires inverting the matrix *R*, which is a 3 × 3 rank-deficient matrix. Indeed, both visual and CDGNSS covariance matrixes are associated to unit vectors and have rank 2. Those contributions are summed up in Equation (16) with two rank-2 matrices, i.e., the attitude related and the magnetometer related matrix, returning a rank 2 *R*. To guarantee invertibility of *R*, linear independent measurements should be used. Therefore, in this derivation, residuals on angles (azimuth *Az* and elevation *El*) **r***_α_* are used instead of residuals on the unit vector **r**:

(20) $$r_{\alpha }=\frac{\partial r_{\alpha }}{\partial r}r=\left[\begin{array}{c} \frac{\partial Az}{\partial u_{}^{c}}\\ \frac{\partial El}{\partial u_{}^{c}} \end{array}\right]r$$

Using azimuth and elevation residuals and their covariance (*R*_α_), the residual and the covariance matrix of Equation (16), i.e., **r** and *R*, become:

(21) $$\begin{array}{c} r_{\alpha }=\underset{J_{\alpha }}{\underbrace{\frac{\partial r_{\alpha }}{\partial r}J}}\delta x\\ R_{\alpha }=\frac{\partial r_{\alpha }}{\partial r}R{\frac{\partial r_{\alpha }}{\partial r}}^{T} \end{array}$$

Following the LM approach, described in Section 2, a set of *k* residuals is used to derive the unknown of the problem. Naming ${\left(R_{\alpha }\right)}_{j}$ and ${\left(J_{\alpha }\right)}_{j}$ the covariance and measurement matrixes related to the *j*-th angular residual vector, i.e., ${\left(r_{\alpha }\right)}_{j}$, the covariance on the unknown vector can be derived as:

(22) $$\mathrm{cov}\left(\delta x\right)={\left({\left[\begin{array}{c} {\left(J_{\alpha }\right)}_{1}\\ {\left(J_{\alpha }\right)}_{2}\\ \vdots \\ {\left(J_{\alpha }\right)}_{k} \end{array}\right]}^{T}{\left[\begin{array}{cccc} {\left(R_{\alpha }\right)}_{1} & 0_{3} & 0_{3} & 0\\ 0_{3} & {\left(R_{\alpha }\right)}_{2} & 0_{3} & 0_{3}\\ 0_{3} & 0_{3} & \ddots & 0_{3}\\ 0_{3} & 0_{3} & 0_{3} & {\left(R_{\alpha }\right)}_{k} \end{array}\right]}^{-1}\left[\begin{array}{c} {\left(J_{\alpha }\right)}_{1}\\ {\left(J_{\alpha }\right)}_{2}\\ \vdots \\ {\left(J_{\alpha }\right)}_{k} \end{array}\right]\right)}^{-1}$$

### 3.1. Error Budget Prediction

**Figure 3:**
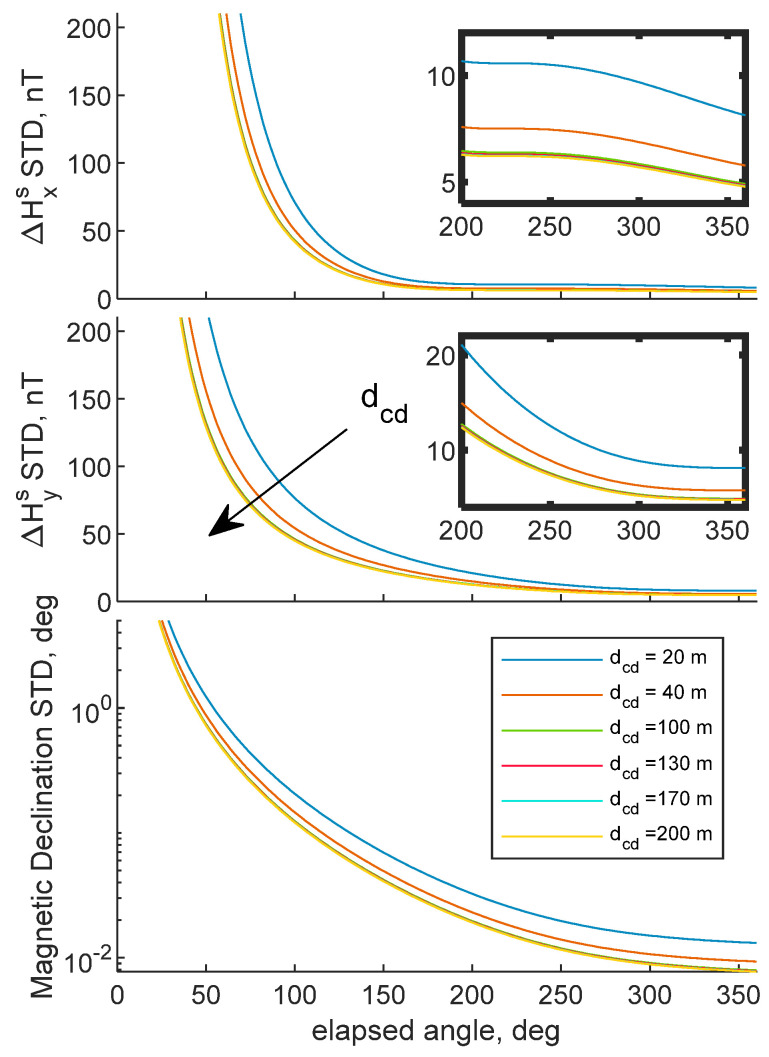
Predicted bias STDs as a function of the rotation performed by the chief UAV. Varying *d_cd_*. *ψ*_0_ = 90°. Zoomed $\Delta H_{x}^{s}$ and $\Delta H_{y}^{s}$ are reported in bolded borders’ rectangles. CDGNSS, horizontal angle, camera and magnetometer STDs are reported in 1.

**Figure 4:**
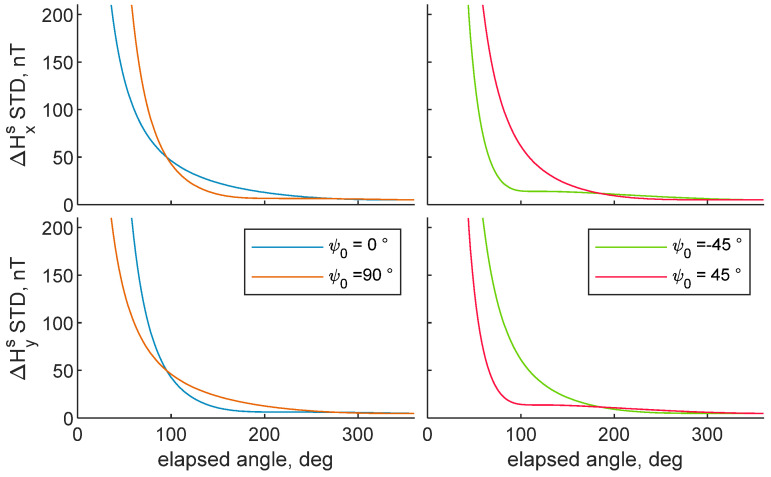
Predicted bias STDs as a function of the rotation performed by the chief UAV. Varying *ψ*_0_. *d_cd_* = 100 m. CDGNSS, horizontal angle, camera and magnetometer STDs are reported in 1.

**Figure 5:**
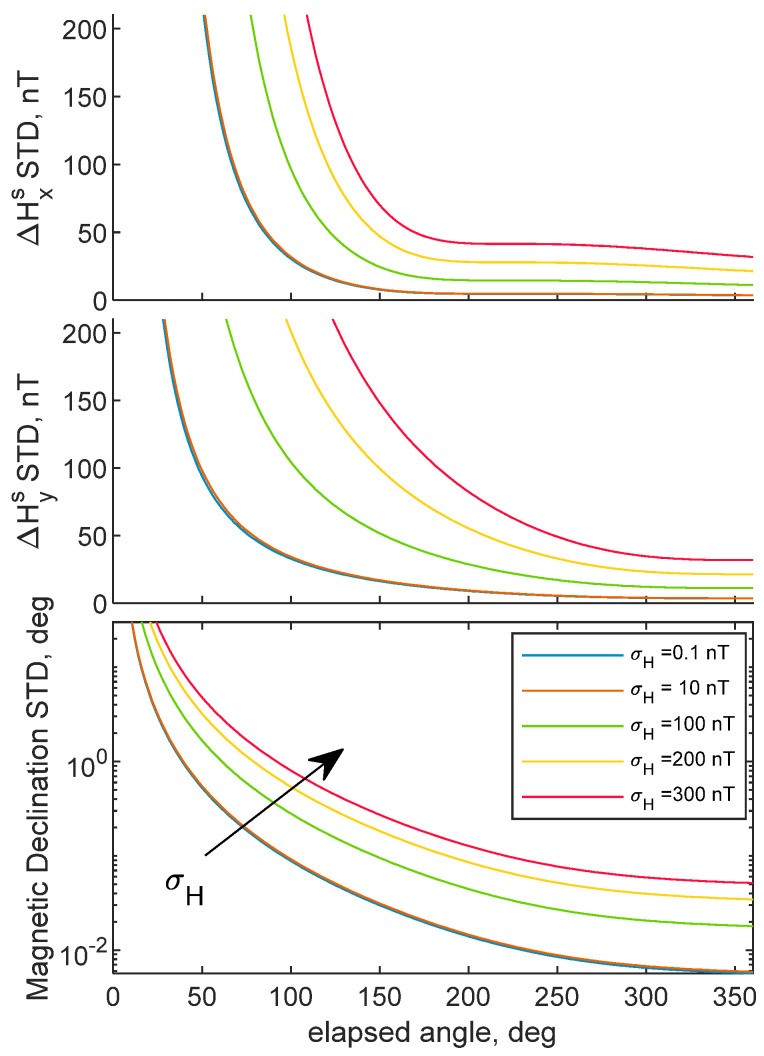
Predicted bias STDs as a function of the rotation performed by the chief UAV. Varying magnetometer STD, i.e., σ*_H_*. *ψ*_0_ = 90°and *d_cd_* = 100 m. CDGNSS, horizontal angle and camera STDs are reported in 1.

The proposed methodology can retrieve magnetic biases by isolating internal disturbance constants in BRF from external magnetic disturbances. Correct estimation of internal magnetic biases requires rotating the UAV with the aim of creating spatial diversity of magnetic field measurements in BRF. An ideal trajectory to fly to correctly estimate magnetic bias consists in rotating the chief UAV along its down body axis. On the other hand, the deputy must fly along a circle with a constant radius with the aim of being always in the chief’s camera FOV. This section aims at evaluating the theoretical estimation accuracy of the problem’s unknowns, using Equation (22). The chief UAV is assumed to be rotating along its down axis by continuously changing its heading. Results show the biases STD as a function of the rotation angle covered by the UAV, from 0° to 360°. Camera, CDGNSS, magnetometers and horizontal attitude angles STD are reported in Table 1. Typical uncertainty values of cameras embarked on UAVs have been used, whereas CDGNSS and horizontal angular accuracy are assumed equal to those obtained by CDGNSS processing and UAV navigation system. Magnetometer resolution has been assumed equal to 10 nT, which is within the range of magneto-resistive sensors [22]. Several configurations have been analyzed. Magnetic bias accuracy as a function of the chief–deputy distance, i.e., *d_cd_*, and the initial heading angle of the chief vehicle, i.e., *ψ*_0_, are reported in Figure 3 and Figure 4. Whereas STDs as a function of magnetometer resolution are highlighted in Figure 5. For the sake of visualization, Figure 3 provides, for $\Delta H_{x}^{s}$ and $\Delta H_{y}^{s}$ STDs, a zoom in the range 200° to 360°. Better biases accuracy is provided with a complete 360° turn. However, satisfactory accuracies can be obtained also with a subsegment of the entire 2π angle. As Figure 3 shows, increasing distance allows improving the biases accuracy, due to the reduction in the CDGNSS covariance, as indicated by Equation (17).

In addition, increasing *r* allows reaching an asymptotic value for the internal bias STD with a smaller fraction of the 360 turn. However, the entity of the range-related improvement reduces with *d_cd_*. Indeed, as pointed out in the error budget derivation, magnetic biases STD depends on CDGNSS, camera, magnetometer, and angular accuracy. Range increase makes the CDGNSS covariance reduce, until it becomes less relevant to the other error sources and a lower bound accuracy is encountered, e.g., when *d_cd_ ≈* 200 m.

Magnetometer biases STD as a function of the elapsed angle while varying *ψ*_0_ from −45° to 90° are reported in Figure 4. Variations in the initial heading do not alter the external bias STD, thus it is not reported in the figure, for the sake of brevity. Conversely, it modifies $\Delta H_{x}^{s}$ and $\Delta H_{y}^{s}$ curves. Indeed, more observability is provided to the internal bias component, which is almost parallel to the magnetic field. This behavior is confirmed by Figure 3, where $\Delta H_{x}^{s}$ converges after 180°, because the $H_{x}^{s}$ direction covers a symmetric interval around the north axis. Figure 4 shows 90° gap couples, i.e., 0° to 90°, −45° to 45°. These couples show an opposite behavior in terms of $\Delta H_{x}^{s}$ and $\Delta H_{y}^{s}$ STDs, i.e., $\Delta H_{x}^{s}$ and $\Delta H_{y}^{s}$ curves at *ψ*_0_, are equal to $\Delta H_{y}^{s}$ and $\Delta H_{x}^{s}$ at *ψ*_0_ + 90°. For the couple 0°–90°, when *ψ*_0_ = 90°, $H_{y}^{s}$ is parallel to the north direction, i.e., the direction along which the magnetic field is the highest, when the rotation begins, and therefore more observable, whereas the opposite configuration, i.e., $H_{x}^{s}$ parallel to the north direction when the rotation starts, holds when *ψ*_0_ = 0°. In the case *ψ*_0_ = −45°, convergence of $\Delta H_{x}^{s}$ is encountered after 90° turn, because it offers a symmetric variation of $H_{x}^{s}$ around the north axis. The opposite behavior, i.e., $\Delta H_{y}^{s}$ converges after 90° turn, is encountered when *ψ*_0_ = 45°. Therefore, magnetic bias convergence can be encountered also by covering an angle that is less than 180° degree, if the vehicle is rotated to make one of the two magnetometer horizontal axes of the vehicle swap an angle symmetric with respect to the direction of the magnetic field, i.e., the north direction. In this case, good observability is provided for the biases associated to the axis whose rotation satisfies that condition, at the expense of the bias error along the other horizontal magnetometer axis.

Figure 5 shows the error budget results as a function of magnetometer STD. Typical values of MEMS magnetometers mounted on conventional drones, i.e., with σ*_H_* up to 200 nT, have been considered.

No significative changes in magnetometer biases STD are obtained when using a more accurate magnetometer, e.g., with σ*_H_* = 0.1 nT. than the one used in Figure 3 and Figure 4. This is due to a covariance lower bound imposed by the other covariance sources reported in Equation (16), whose relative importance increases with respect to the magnetometer covariance, for values of σ*_H_* smaller than 10 nT. Using higher values of σ*_H_*, in the orders of hundreds of nT, increases the asymptotic value, i.e., the minimum accuracy obtained after a 360° turn, with a quasi-linear behavior in $\Delta H_{x}^{s}$ and $\Delta H_{y}^{s}$ STDs. In addition, σ*_H_* increase makes the asymptotic condition to be reached with a highest elapsed angle, equal to 180° for $\Delta H_{x}^{s}$ when σ*_H_* = 300 nT. Figure 5 suggests sub-degree precision in magnetic declination can be obtained with a 360 turn when classic magnetometer mounted on conventional drones are used. Accuracy in the order of tens of nT is obtained for internal bias estimation. The figure suggests that better performance in magnetometer biases estimation can be obtained with more accurate magnetometer sensors.

## 4. Experimental Setup and Flight Scenario

### 4.1. Experimental Setup

**Figure 6:**
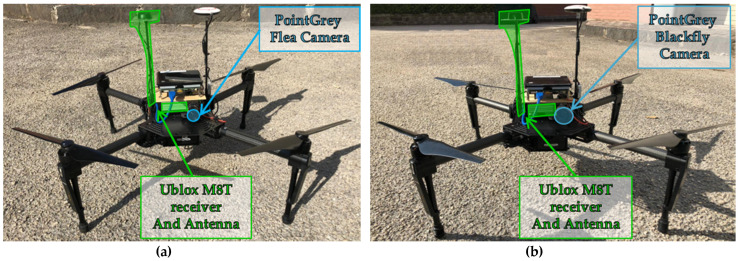
Eagle (**a**) and Athena (**b**) setups, showing camera and GNSS receiver and antenna.

The flight test was carried out using a customized version of the DJI M100 quadrotor as chief, which has been given the name “Eagle”. Such a vehicle is equipped with an onboard computer (Intel NUC with i7 CPU), an additional GNSS single frequency receiver (uBlox LEA-M8T), an auxiliary GNSS antenna and a CMOS camera (PointGrey Flea FL3-U3-20E4C-C) collecting data at a frequency of around 12 Hz. Camera characteristics are shown in Table 2. The deputy vehicle is another customized version of the DJI M100 quadrotor, this time equipped with the very same onboard computer with an i5 CPU, and a uBlox LEA-M8T GNSS single frequency receiver and antenna. The deputy, named “Athena”, is also equipped with a camera relying on the CCD (PointGrey BlackFly BFLY-U3-50H5C-C) as detector. Both deputy and chief are shown in Figure 6. In addition, a ground fixed GNSS antenna and receiver (Trimble AV59 and BD960) have been used as a Ground Control Point for assessing the performance of the proposed methodology, as further discussed in Section 5.

### 4.2. Flight Scenario

The experimental campaign, which took place in Acerra (NA), Italy in December 2019, consisted of two flights. At this location and date, the magnetic declination predicted by the International Geomagnetic Reference Field is 3.39° [23]. Due to the relative geometry of the two UAVs, including relative turns and large heading variations, the second flight has been chosen to carry out magnetometer bias estimation. During this flight, which lasted around 11 min, chief GNSS, magnetometers and IMU data were, respectively, collected at 1 Hz, 10 Hz and about 137 Hz.

**Figure 7:**
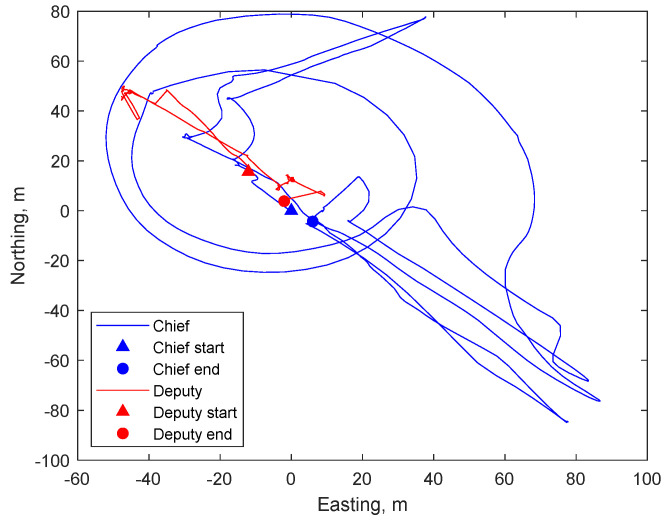
Graphic illustration of chief and deputy trajectory in easting and northing as computed with GNSS data.

**Figure 8:**
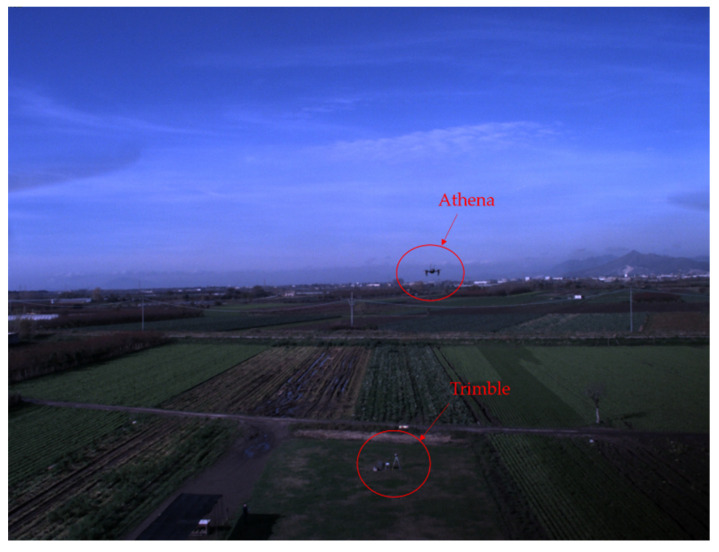
Example of chief-taken frame where both deputy (Athena) and ground-based GNSS antenna (Trimble) are visible.

The flight has been characterized by maneuvers to keep the two UAVs facing each other, and this has been achieved by rotating the chief around the deputy as it is easily noticeable in Figure 7, where easting and northing of both UAVs as computed with their GNSS data (also indicating the starting and ending points) are shown. The above-ground flight altitude of both UAVs has not varied significantly, as a matter of fact, its mean values, as evaluated by GNSS, are around 16.0 m for Athena and 16.8 m for Eagle. A video sequence, compressed and accelerated (2x) to reduce file dimensions, composed of chief-taken frames in a fraction of the flight test, can be found in Video S1 of Supplementary Materials. A chief-taken image showing both Athena and the ground-based GNSS (Trimble) antenna is shown in Figure 8, where both targets can be easily detected by eye.

**Figure 9:**
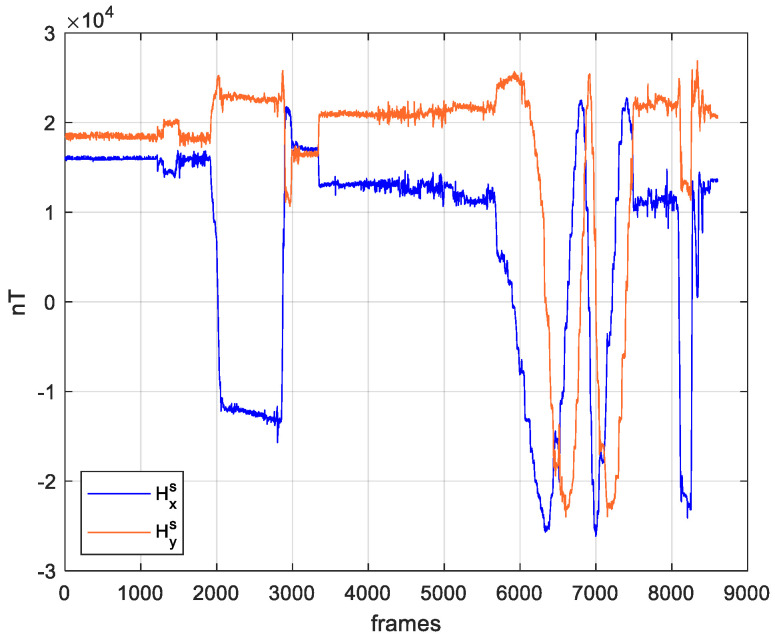
Horizontal BSRF components of the magnetometer measurements in nT as provided by the DJI Onboard SDK interface.

The magnetometers output during the flight is shown in Figure 9, where both x and y components of the sensed Earth magnetic field in BSRF are depicted. Such quantities are provided by the chief autopilot through the DJI Onboard SDK interface, in (non-better specified) “normalized” magnetic unit (based on DJI documentation, magnetic measurements are normalized so that the norm is included in the range between 1000 and 2000).

As a reference, the norm of the Earth magnetic field vector at the flight location and date is equal to 46,432 nT, as estimated from [23]. An analysis on a static portion of the data acquisition, i.e., including the first 60 s, shows a scale factor of about 29, which is used in the following to convert DJI normalized units to nT. This scale factor has been obtained by dividing the norm of the predicted magnetic field in nT by the norm of the measured magnetic field (in DJI normalized units) in static configuration.

## 5. Results

This section presents the results of the magnetic calibration procedure. The iteration process, which leads to the estimation of both internal biases and external magnetic field declination, starts by selecting a set of camera frames as inputs. This selection is reported in Section 5.1, whereas magnetic biases estimation results and their predicted STDs are reported in Section 5.2 and Section 5.3.

### 5.1. Frame Selection

Frames used in the analysis have been selected based on constraints imposed on chief-to-deputy distance, which is kept larger than 30 m to improve the accuracy of the CDGNSS-based LOS unit vector in NED, and on the absolute value of chief yaw rate and pitch and roll angles (as estimated by its onboard navigation system). To avoid too fast heading variations, which may pose challenges due to the low magnetometer bandwidth and the residual data synchronization errors, the yaw rate is requested to be smaller than 1.5°/s, while maximum pitch and roll angles of 6.5° are considered so that the small angles assumption holds.

**Figure 10:**
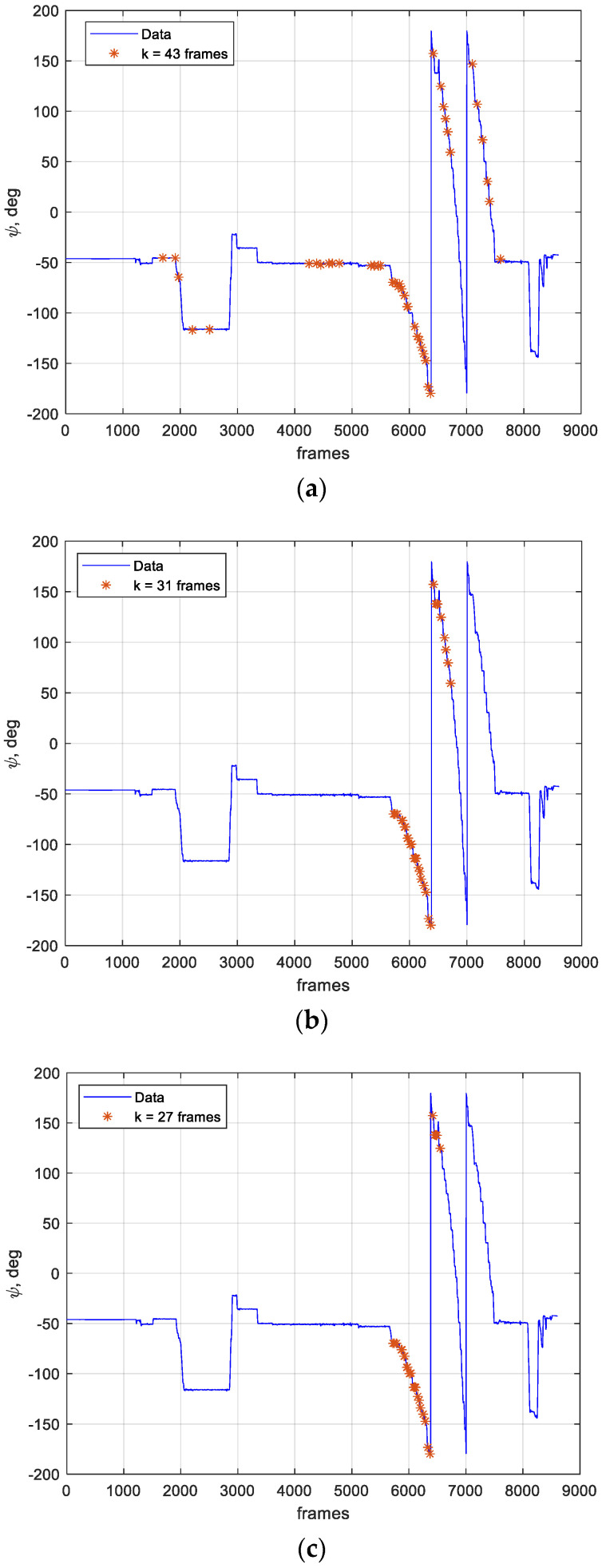
Chief heading variation during the flight resampled in camera time with frames chosen for computation, orange asterisks. (**a**) shows frames chosen for the whole flight case analysis, (**b**) and (**c**) show frames chosen for subsets 1 and 2, respectively.

After applying these constraints, frames have been organized for covering three different flight portions identified as “Whole Flight”, in which all frames compliant with the aforementioned requirements have been used, and “subset 1” and “subset 2”, in which selected frames are extracted from smaller intervals. These regions are shown in Figure 10, where the heading angle behavior, as estimated by the onboard navigation filter (ψ) and resampled in camera time, is depicted for the flight duration along with the frames selected for each computation, in a total of *k*.

Each flight portion case was obtained from the previous ones by reducing the selected frames interval. It must be noticed that the imposition of the CDGNSS-based baseline, chief yaw rate and pitch and roll angles constraints represents a strong limitation in the frames selection process. In the subset 1 case, the heading angle changes from −69.8° to 59.4°, while in the subset 2 case, it varies from −69.8° to 124.7°. The whole flight case is the one involving the greater number of frames, 43, which reduces, respectively, to 31 and 27 for the subset cases, from higher to lower heading rotations.

Mean and extreme values of chief yaw rate (Ω*_z_*) and chief–deputy CDGNSS-based baseline in terms of the norm of the unit vector ${\left.u_{}^{c}\right|}_{cdgnss}$ for each of these analyzed cases are reported in Table 3. Table 4 includes maximum absolute values and mean values of pitch and roll angles.

### 5.2. Biases Estimation Results

Convergence criteria thresholds of the LM procedure (i.e., ε_1,_ ε_2_ and ε_3_) have been set to 10^−8^, 10^−4^ and 10^−16^. The initial guesses of the unknown parameters have been set to zero, for the internal magnetic bias components in BSRF, and to the true value of magnetic declination (3.39°) for the *d_m_* angle. The initial value of the damping parameter (*λ*_0_) has been set to 10^−4^.

The quality of the iterative procedure is mainly assessed by the value reached by the residual chi-squared function at the iteration end divided by the total number of frames involved in the computation, referred to as $\chi_{k}^{2}$. Such quantity is listed in Table 5 for each case, along with the type of convergence achieved, reported as type 1, 2 or 3 as in reading order from (11), and the number of iterations needed for such convergence to succeed, *it*. In all the cases, a value of $\chi_{k}^{2}$ of the 10^−3^ order is obtained after a quite small number of total iterative steps, thus suggesting satisfactory convergence conditions. The estimated magnetic biases are listed in Table 6, where the internal magnetic bias horizontal components in BSRF are reported in normalized magnetic units.

With the aim of better understanding the differences among the different solutions and the statistical significance of the results presented in Table 6, the solution uncertainty derived based on the previously presented error budget is reported in Table 7. Differently from the results shown in Section 3.1, $\Delta H_{x}^{s}$ and $\Delta H_{y}^{s}$ STD are reported in normalized units for the sake of consistency with the results in Table 6. The values reported in Table 7 have been derived using only the measurements relevant to the frames identified in Section 5.1, for the whole flight and the subset cases. Camera and horizontal angle STDs have been assumed equal to 0.1° and 1°, respectively. Magnetometer STD has been estimated from the measured components of the magnetic field in static configuration, which holds in the first 60 s of the flight. STD estimated on normalized components have been converted in nT by using the actual intensity of the magnetic field in nT reported in [23]. This procedure yields a value of about 150 nT for the magnetometer STDs on the horizontal components. To consider the slightly non-horizontal configuration (i.e., maximum pitch and roll angles equal to 6.5°), the estimated horizontal magnetometer STD, has been amplified by considering an additional noise obtained combining the vertical magnetic field with the standard deviation of roll and pitch angles.

Comparing Table 6 and Table 7 highlights that variations of estimated biases are consistent with 1σ bounds in each case. As expected, the STD values increase as the number of frames reduces from the whole flight to the subset 2 case. However, results achieved with “whole flight” and “subset 1” dataset differ only slightly in terms of estimated biases and STD, thus demonstrating that the chief horizontal attitude calibration can be performed by avoiding the sampling of frames from the whole flight and, instead, exploiting a reduced number of frames which belong to a specific flight dynamic portion.

### 5.3. Pointing Error Analysis

The quality of the magnetic heading calibration procedure, resulting from the estimated magnetic biases and declination listed in Table 6, has been analyzed by performing a pointing error analysis, following the same approach of [24] and using an ad hoc selected control point (CP). The unit vector between the chief UAV and the CP is computed in NED using CDGNSS processing, and the azimuth angle is extracted to be used as (attitude independent) reference measurement (*Az_ref_*). The azimuth angle is then evaluated using images and the estimated chief attitude angles. In particular, the pixels coordinates corresponding to the control point location in chief-taken images are first used to compute the chief-to-CP unit vector in CRF. This unit vector is then transformed in NED by using $M_{b}^{c}$ and the NED-BRF rotation matrix evaluated by using three different heading angles: the calibrated and non-calibrated magnetic ones, which lead, respectively, to $Az_{m,C}$ and $Az_{m,NC}$, and the heading angle as estimated by the onboard DJI filter, leading to $Az_{DJI}$. The pointing error (i.e., Δ*Az*) is the difference between these angles and the reference one, i.e., *Az_ref_*, and it can thus be computed and used for performance assessment. The uncertainty of the pointing error estimate is evaluated by summing up the CDGNSS covariance, expressed in angular quantities via Equation (20) and the camera angular uncertainty. These quantities are representative of the error on *Az_ref_* and camera estimated quantities (i.e., *Az_m,C_*, *Az_m,NC_* and *Az_DJI_*), respectively.

**Figure 11:**
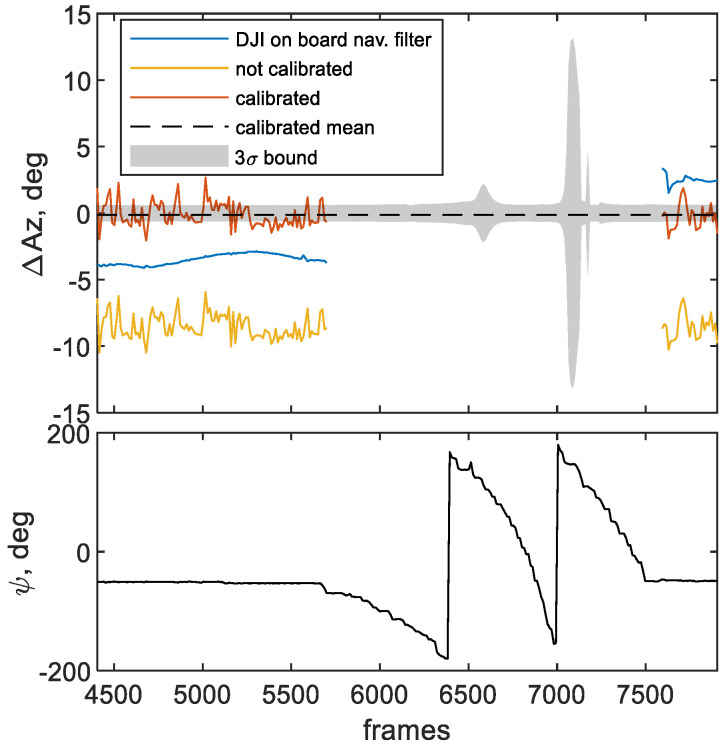
Pointing analysis results of the whole flight case. Pointing error is shown in the above panel, along with the 3σ bound and the calibrated azimuth error mean (dashed black line). DJI filter-estimated heading angle is shown in the below panel. Both figures are referred to frame intervals where the Trimble target is visible.

Results of such analysis are shown in Figure 11 for the whole flight case when the Trimble ground-based antenna is used as ground control point (GCP). In the first panel of the figure the pointing errors computed with *Az_m,C_*, *Az_m,NC_* and *Az_DJI_* are, respectively, shown in orange, yellow and blue. While the second panel, shows the heading angle ψ as computed by the DJI filter. It must be mentioned that pointing errors can only be computed with respect to frames where the Trimble antenna can be easily detected. This occurs in two main frame intervals, as Figure 11 shows.

The calibrated pointing error performance appears to be significantly improved with respect to both non-calibrated and DJI-based heading estimates, thus clearly proving the effectiveness of the proposed calibration methodology. This can be inferred by considering the statistics of each pointing error, mainly in terms of mean and root mean squared error (RMS). As a matter of fact, the calibrated mean value (−0.17°), also shown in the figure as a dashed black line, is remarkably smaller than the non-calibrated (−8.5°) and DJI-based ones (−2.3°). This trend is confirmed by the calibrated RMS (0.95°), which is again smaller than the non-calibrated (8.6°) and DJI-based ones (3.3°). Furthermore, the mean value of the pointing error for the calibrated heading appears to be well contained within the 3σ bound region for the benchmark, highlighted in grey in Figure 11. It is worth noting that pointing errors of the DJI onboard filter change in the initial and final phases of the flight even though the heading angle is similar, because of the filter dependency on the experimented dynamics.

It is also worth mentioning that performing calibration with the method in [16], which does not allow estimating the external bias, results in a pointing accuracy mean error equal to −2.7° in the whole flight case.

**Figure 12:**
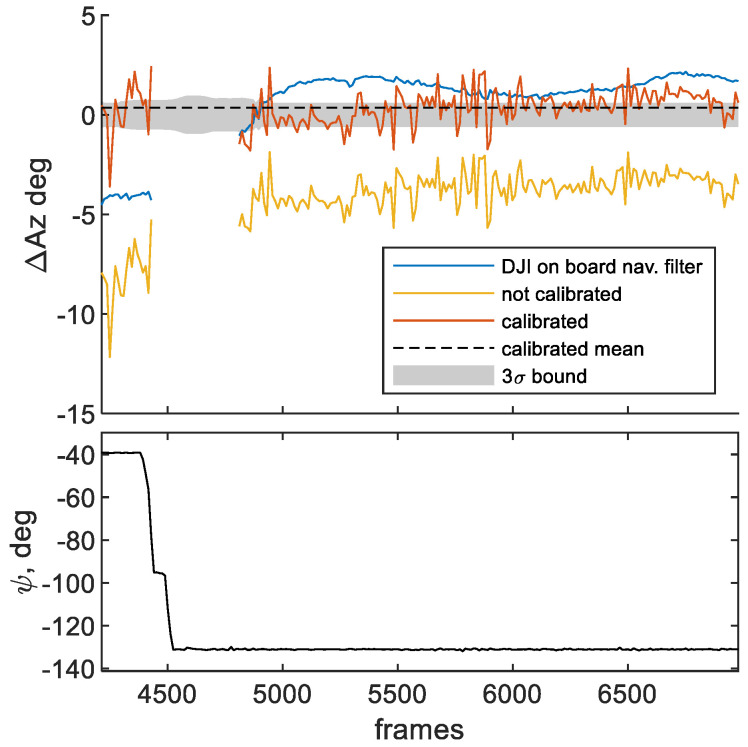
Pointing analysis results on Flight 1. Athena has been used as CP. Calibrated and uncalibrated azimuth have been obtained with magnetometer biases estimated along the whole flight 2 and reported in Table 6.

To further assess the quality of the calibration procedure, the pointing analysis is also performed considering the other flight experiment carried out during the same day (flight 1), and using the other drone (Athena) as an airborne control point. As before, *Az_m,C_* and *Az_m,NC_* have been estimated by using calibrated and un-calibrated heading angles (Equations (3) and (4), respectively) with magnetometer biases set as those reported in Table 6, for the whole flight case. Pointing analysis results are shown in Figure 12. The difference between *Az_m,C_* and *Az_ref_*, which is depicted in orange, has a mean that is well within the 3σ bound. In addition, analyzing the link between the pointing results and the heading variation, it is possible to verify that pointing accuracy is independent from heading for the calibrated solution. Indeed, azimuth error results to be heading dependent, for other cases (*Az_m,NC_* and *Az_DJI_*), thus demonstrating a worse compensation of onboard magnetic biases.

## 6. Conclusions

This paper presented a calibration technique designed to simultaneously estimate the onboard horizontal magnetic biases and the external magnetic declination. The approach is based on the exploitation of cooperative navigation with one (or more) deputy UAV and on the usage of drone-to-drone visual and CDGNSS measurements. The ad hoc derived error budget showed that, besides the magnetometer noise, the relative flight geometry (i.e., the distance between UAVs and the interval of heading angles in which cooperative measurements are available), has a strong impact on the accuracy of the calibration procedure. Specifically, accurate estimation of magnetic declination, at the sub-degree level, can be obtained with relatively short baselines and using commercial hardware. Experimental results were also shown, which are promising and further corroborate the potential of the developed approach. In fact, the magnetic calibration results are consistent with error budget predictions. Moreover, a pointing analysis based on both one ground and one airborne control point demonstrated the possibility to achieve an angular pointing accuracy below one degree exploiting magnetic heading estimates based on the proposed calibration procedure. Such an accuracy level has been demonstrated to be much better than the one achievable using either the non-calibrated magnetic heading, or the attitude estimate of the DJI onboard navigation filter, which resulted in root mean square errors of about nine and three degrees, respectively.

Based on these results, future research is foreseen in different directions. First, the cooperative method will be extended towards full 3D magnetic calibration. This requires proper attitude variations (in roll and pitch) to enhance the observability of vertical biases. In this scenario, an IMU/magnetometer independent estimation of the full attitude state can be guaranteed by exploiting two or more deputies as reported in [23]. Consistently with these developments, the potential of the technique towards UAV-based magnetometry will be explored, also taking advantage of high sensitivity magnetometers embarked as mission payloads.

## Figures and Tables

**Table 1:** Simulation parameter for error budget prediction.

	Variable	Value
Camera STD	σ*_cam_*	0.1°
CDGNSS STD	$\Sigma_{cdgnss}$	[0.05, 0.05, 0.15]
Magnetometer STD	$\sigma_{H}=\delta H_{x}^{s}=\delta H_{y}^{s}$	10 nT
Horizontal angles STD	*ρ_N_, ρ_E_*	1°

**Table 2:** Chief (Eagle) camera characteristics.

Eagle Camera Characteristics	
Resolution in pixels	1600 × 1200
Max frame rate	59 fps
Focal length	8 mm
IFOV	0.03°

**Table 3:** Chief yaw rate (Ω*z*) and chief–deputy CDGNSS-evaluated range absolute value $\left(\left|{\left.u_{}^{c}\right|}_{cdgnss}\right|\right)$ extreme and mean values in the analyzed cases.

Cases	Ω*_z_*, °/s		$\left|{\left.u_{}^{c}\right|}_{cdgnss}\right|,\;m$	
	Max	Mean	Min	Mean
Whole flight	1.4	0.58	30.1	60.7
Subset 1	1.4	0.57	47.9	66.4
Subset 2	1.4	0.59	62.7	68.2

**Table 4:** Chief pitch (*ϑ*) and roll (*φ*) angles as evaluated by onboard DJI filter maximum and mean values in the analyzed cases.

Cases	*φ*, Deg		*Θ*, Deg	
	Max	Mean	Max	Mean
Whole flight	6.4	3.2	5.6	2.3
Subset 1	6.4	4.0	5.6	2.1
Subset 2	6.4	3.9	5.6	2.0

**Table 5:** LM procedure convergence results.

Cases	*it*	$\chi_{k}^{2}$	Convergence Type
Whole flight	3	2.4 × 10^−3^	‘1’
Subset 1	4	1.6 × 10^−3^	‘1’ and ’2’
Subset 2	4	1.3 × 10^−3^	‘1’ and ’2’

**Table 6:** Results of LM iterations in terms of estimated magnetic biases.

Cases	$\Delta H_{x}^{s}$	$\Delta H_{y}^{s}$	$d_{m},\;\mathrm{Deg}$
Whole flight	−45.84	43.18	7.58
Subset 1	−44.04	41.89	7.69
Subset 2	−38.40	50.89	8.25

**Table 7:** Error budget’s expected STDs.

Cases	$\Delta H_{x}^{s}$	$\Delta H_{y}^{s}$	$d_{m},\;\mathrm{Deg}$
Whole flight	6.86	7.45	0.37
Subset 1	7.44	12.85	0.51
Subset 2	9.80	16.51	0.80

## Data Availability

Data sharing is not applicable to this article.

## Supplementary Materials

The following are available online at https://www.mdpi.com/article/10.3390/s21113582/s1. Video S1: Compressed and accelerated (2x) video sequence of the deputy observed by the chief camera during the 360° turn.

## References

[1] Arnold R.D., Yamaguchi H., Tanaka T. (2018). Search and rescue with autonomous flying robots through behavior-based cooperative intelligence. J. Int. Humanit. Action.

[2] Pizetta I.H.B., Brandão A.S., Sarcinelli-Filho M. Cooperative quadrotors carrying a suspended load. Proceedings of the 2016 International Conference on Unmanned Aircraft Systems (ICUAS).

[3] Kopeikin A., Russell C., Trainor H., Rivera A., Jones T., Baumgartner B., Manjunath P., Heider S., Surdu T., Galea M. Designing and Flight-Testing a Swarm of Small UAS to Assist Post-Nuclear Blast Forensics. Proceedings of the 2020 International Conference on Unmanned Aircraft Systems (ICUAS).

[4] Martínez-de-Dios J.R., Merino L., Ollero A., Ribeiro L.M., Viegas X. (2007). Multi-UAV Experiments: Application to Forest Fires.

[5] Lu G., Cannon M.E. (1994). Attitude determination using a multi-antenna GPS system for hydrographic applications. Mar. Geod..

[6] Fang J., Sun H., Cao J., Zhang X., Tao Y. (2011). A Novel Calibration Method of Magnetic Compass Based on Ellipsoid Fitting. IEEE Trans. Instrum. Meas..

[7] Vasconcelos J.F., Elkaim G., Silvestre C., Oliveira P., Cardeira B. (2011). Geometric Approach to Strapdown Magnetometer Calibration in Sensor Frame. IEEE Trans. Aerosp. Electron. Syst..

[8] Chodnicki M., Mazur M., Nowakowski M., Kowaleczko G. Algorithms for the Detection and Compensation Interferance of Magnetic Field Measurement. Proceedings of the 2018 5th IEEE International Workshop on Metrology for AeroSpace (MetroAeroSpace).

[9] Wu H., Pei X., Li J., Gao H., Bai Y. (2020). An improved magnetometer calibration and compensation method based on Levenberg–Marquardt algorithm for multi-rotor unmanned aerial vehicle. Meas. Control.

[10] Lee T.N., Canciani A.J. (2020). MagSLAM: Aerial simultaneous localization and mapping using Earth’s magnetic anomaly field. J. Navig..

[11] Funaki M., Higashino S.-I., Sakanaka S., Iwata N., Nakamura N., Hirasawa N., Obara N., Kuwabara M. (2014). Small unmanned aerial vehicles for aeromagnetic surveys and their flights in the South Shetland Islands, Antarctica. Polar Sci..

[12] Schmidt V., Becken M., Schmalzl J. (2020). A UAV-borne magnetic survey for archaeological prospection of a Celtic burial site. First Brea..

[13] Jackisch R., Madriz Y., Zimmermann R., Pirttijärvi M., Saartenoja A., Heincke B.H., Salmirinne H., Kujasalo J.-P., Andreani L., Gloaguen R. (2019). Drone-Borne Hyperspectral and Magnetic Data Integration: Otanmäki Fe-Ti-V Deposit in Finland. Remote Sens..

[14] Le Maire P., Bertrand L., Munschy M., Diraison M., Géraud Y. (2020). Aerial magnetic mapping with an unmanned aerial vehicle and a fluxgate magnetometer: A new method for rapid mapping and upscaling from the field to regional scale. Geophys. Prospect..

[15] Vetrella A.R., Fasano G., Accardo D. (2019). Attitude estimation for cooperating UAVs based on tight integration of GNSS and vision measurements. Aerosp. Sci. Technol..

[16] Vetrella A.R., Causa F., Renga A., Fasano G., Accardo D., Grassi M. (2019). Multi-UAV Carrier Phase Differential GPS and Vision-based Sensing for High Accuracy Attitude Estimation. J. Intell. Robot. Syst..

[17] Opromolla R., Esposito G., Fasano G. In-flight estimation of magnetic biases on board of small UAVs exploiting cooperation. Proceedings of the 2019 IEEE 5th International Workshop on Metrology for AeroSpace (MetroAeroSpace).

[18] Opromolla R. (2020). Magnetometer Calibration for Small Unmanned Aerial Vehicles Using Cooperative Flight Data. Sensors.

[19] Gavin H. The Levenberg–Marquardt Method for Nonlinear Least Squares Curve-Fitting Problems. http://people.duke.edu/~%7B%7Dhpgavin/ce281/lm.pdf.

[20] Opromolla R., Inchingolo G., Fasano G. (2019). Airborne Visual Detection and Tracking of Cooperative UAVs Exploiting Deep Learning. Sensors.

[21] Furgale P., Rehder J., Siegwart R. Unified temporal and spatial calibration for multi-sensor systems. Proceedings of the 2013 IEEE/RSJ International Conference on Intelligent Robots and Systems.

[22] (2018). Honeywell Magnetic Sensor Product Catalog. https://aerospace.honeywell.com/content/dam/aero/en-us/documents/learn/products/sensors/product-catalog/Sensors_Product_Catalog.pdf.

[23] National Center for Environmental Information Magnetic Declination Calculator. https//www.ngdc.noaa.gov/geomag/calculators/magcalc.shtml.

[24] Vetrella A., Fasano G., Accardo D., Moccia A. (2016). Differential GNSS and Vision-Based Tracking to Improve Navigation Performance in Cooperative Multi-UAV Systems. Sensors.

